# Meta-analysis of drug-related deaths soon after release from prison

**DOI:** 10.1111/j.1360-0443.2010.02990.x

**Published:** 2010-09

**Authors:** Elizabeth L C Merrall, Azar Kariminia, Ingrid A Binswanger, Michael S Hobbs, Michael Farrell, John Marsden, Sharon J Hutchinson, Sheila M Bird

**Affiliations:** 1 MRC Biostatistics UnitCambridge, UK; 2 National Centre in HIV Epidemiology and Clinical ResearchSydney, NSW, Australia; 3 Division of General Internal Medicine, University of Colorado at Denver School of MedicineDenver, CO, USA; 4 School of Population Health, The University of Western AustraliaCrawley, WA, Australia; 5 National Addiction Centre, Division of Psychological Medicine and Psychiatry, Institute of Psychiatry, King's College LondonLondon, UK; 6 Health Protection ScotlandGlasgow, UK; 7 Department of Statistics and Modelling Science, Strathclyde UniversityGlasgow, UK; 8 Denver Health Medical CenterDenver, CO, USA

**Keywords:** street drugs, substance-related disorders, mortality, overdose, prisons, prisoners, meta-analysis

## Abstract

**Aims:**

The transition from prison back into the community is particularly hazardous for drug-using offenders whose tolerance for heroin has been reduced by imprisonment. Studies have indicated an increased risk of drug-related death soon after release from prison, particularly in the first 2 weeks. For precise, up-to-date understanding of these risks, a meta-analysis was conducted on the risk of drug-related death in weeks 1 + 2 and 3 + 4 compared with later 2-week periods in the first 12 weeks after release from prison.

**Methods:**

English-language studies were identified that followed up adult prisoners for mortality from time of index release for at least 12 weeks. Six studies from six prison systems met the inclusion criteria and relevant data were extracted independently.

**Results:**

These studies contributed a total of 69 093 person-years and 1033 deaths in the first 12 weeks after release, of which 612 were drug-related. A three- to eightfold increased risk of drug-related death was found when comparing weeks 1 + 2 with weeks 3–12, with notable heterogeneity between countries: United Kingdom, 7.5 (95% CI: 5.7–9.9); Australia, 4.0 (95% CI: 3.4–4.8); Washington State, USA, 8.4 (95% CI: 5.0–14.2) and New Mexico State, USA, 3.1 (95% CI: 1.3–7.1). Comparing weeks 3 + 4 with weeks 5–12, the pooled relative risk was: 1.7 (95% CI: 1.3–2.2).

**Conclusions:**

These findings confirm that there is an increased risk of drug-related death during the first 2 weeks after release from prison and that the risk remains elevated up to at least the fourth week.

## INTRODUCTION

For drug-using offenders, imprisonment can enforce a substantial reduction in drug use and, as a result, drug tolerance [1]. Seaman *et al*. [2] first showed that, for a cohort of 316 male human immunodeficiency virus (HIV)-infected injecting drug-users in Scotland, the relative risk (RR) of overdose death was eight times higher [95% confidence interval (CI): 1.5–39.1] in the first 2 weeks after release from prison compared with subsequent 2-week periods (weeks 3–12). Bird & Hutchinson [3] reinforced these findings in their later, Scottish study of 19 486 male index releases (RR: 7.4, 95% CI: 3.3–16.3). By not requiring knowledge of prisoners' injector status, this study ascertained all drug-related deaths and, hence, avoided reliance on drug-using prisoners' willingness to self-identify.

Similarly large studies of prisoners' mortality post-release have since been conducted elsewhere, focusing upon the persistence of increased risk in and beyond the first 2 weeks [4–11]. These mortality studies have used database linkage as a practical alternative to more traditional, individually consented prospective cohort studies. Database linkage requires minimal information about eligible ex-prisoners, is less costly and avoids potential biases from opt-ins and dropouts. The research team needs only to specify to a prison service the criteria for eligible releases. The corresponding registrar of deaths can then provide the date and cause of death for any person thus listed. The research team receives a file, without the need for prisoner names, from the prison service with such minimal details as the released prisoner's age, sex, prison and date of release and sentence duration—with which the death information can then be merged.

Index release has been defined variably in the literature. The most recent release from prison is outcome-dependent for those who die and so gives biased ascertainment of deaths [12]. Preferable definitions of index release are: either first release in a defined accrual period (as first release, unlike last release, is independent of 12-week survival status) or all releases in a defined accrual period.

Through meta-analysis [3] of studies of prisoners' mortality published by August 2009, we have summarized the RR of drug-related death (i.e. overdose or accidental poisoning) in the first 2 weeks after release compared with weeks 3–12 after release. To characterize further the decay in risk over time, we have pooled the RR of drug-related death during weeks 1 + 2 and 3 + 4 versus weeks 5–12 after release. This investigation was designed to update, and make more precise, our understanding of the high risk of drug-related death soon after release from prison.

## METHODS

### Search strategy

The MEDLINE (January 1966–August 2009) database was searched using the following combinations of the Medical Subject Headings (MeSH) terms: illicit/street drugs and mortality and prisons/ prisoners; prisoners and mortality/ overdose; prisoners and substance-related disorders and mortality. The following combinations of text words were also used in the MEDLINE database and, additionally, in the Google Scholar™ (2009) search engine: prison, release, overdose, drug; and drug-related deaths, prison, release. These searches were repeated using alternative expressions for prison, namely: jail, gaol, custody and incarceration. The resulting titles and abstracts were then verified for suitable inclusion. The identified studies were scanned for further references and their authors were contacted about published and unpublished studies of which they were aware.

### Inclusion/exclusion criteria

Studies of prisoners' mortality were eligible for inclusion if English-language and:

1. Adult prisoners aged 18–35 years were included in the study (in order to fit the age profile of drug-users in prisons [13]); and follow-up for mortality was from the time of index release for at least 12 weeks. The 12-week follow-up period is sufficiently long to detect variations in risk [3].
2. Numbers of drug-related deaths during weeks 1 + 2, 3 + 4 and 5–12 after release could be extracted from the published literature or by contacting the authors.
3. Drug usage or dependency was not an eligibility criterion for entry into the study. Some individuals at risk of drug-related death after release may have neither self-identified as a drug user nor been diagnosed or detected as a drug user while in prison. Hence, studies restricted to prisons' identified drug users under-ascertain the number of drug-related deaths. Moreover, for this identified subpopulation, the evolution of risk of drug-related death after release may be different. Secondly, the inclusion of non-drug users does not affect the RRs of interest which are essentially the time-adjusted ratio of drug-related deaths.
4. Studies that restrict follow-up to the most recent release are excluded. Use of most recent release introduces numerator bias by excluding the actual amount of time spent at liberty after previous releases. Acceptable definitions of index release are: either first release in a defined accrual period or all releases in a defined accrual period (preferably with allowance for multiplicity).

### Data extraction

Drug-related deaths were defined according to the authors' definitions (see Supporting Information, Appendix S1; details at the end).

In the eligible Scottish, Australian and Washington state studies, the follow-up period started from the date of index-release until the earliest of date of death, subsequent incarceration and end of study, as shown in Table 1. The other eligible studies did not include a censor mechanism for re-incarcerations. However, Bird & Hutchinson [3] demonstrated that, in the absence of this censoring, RRs within 12 weeks of index release were estimated robustly. Moreover, it is non-trivial to implement this censoring in jurisdictions which lack unique prisoner numbers (for example: England and Wales).

**Table 1:** Studies Included in the meta-analysis.

*Study*	*Country*	*Period of release*	*Population*	*Comments*
Bird & Hutchinson 2003 [3]	Scotland, UK	1996–1999 July–December only	19 486 male index releases after 14+ days imprisonment, age at release: 15–35 years.	Index release was first release for a person within a calendar year. A person may have index releases in more than one calendar year. Period at liberty is censored at earlier of date of death or date of first re-incarceration within 12 weeks of index release. Crude drug-related mortality during first 12 weeks post-release was 15.0 per 1000 person-years.
Farrell & Marsden 2005, 2008 [4, 5] (subsumes Singleton *et al*. 2003 [6])	England & Wales, UK	1998–2000	48 771 index releases (36 513 men, 12 258 women), age at release: 15+ years, of whom 49% were aged 15–34 years.	Female releases were oversampled, taken from monthly index database. For men, index releases were taken during 3 months in each year. No consideration given to subsequent incarcerations and so the RRs may have been slightly underestimated. Estimate person-years from no. in sample × days of at-risk period (days halved per deaths). Crude drug-related mortality during the first year after release was 5.2 and 5.9 per 1000 person-years for men and women, respectively. Includes intentional drug-related deaths.
Hobbs *et al*. 2006 [7]	Western Australia, Australia	1995–2001	12 867 index releases (11 303 men, 1564 women), mean age at release: 30.3 years (SD = 9.9) (range = 17–91; with 74% aged 15–35 years).	Additional checks by Hobbs *et al*. led to exclusion of 800 individuals from the 13 667 originally reported on account of deaths in custody, deportation or releases dated after December 2001. Time at risk defined as period from date of first release from prison in the study period until date of death or censoring date (1 January 2004), but excluding subsequent periods of incarceration. Crude drug-related mortality over mean follow-up period of 5.5 years was 1.9 per 1000 person-years.
Kariminia *et al*. 2007 [8]	New South Wales, Australia	1988–2002	85 196 (76 376 men, 8820 women), mean age at release: 30.2 years (SD = 9.1) (range = 18–86; with 77% aged 18–35 years).	Time at risk defined as period between release from prison and death, re-incarceration, or end of study. Crude drug-related mortality over median follow-up period of 7.7 years was 2.9 and 3.5 per 1000 person-years in men and women, respectively. Includes intentional drug-related deaths.
Binswanger *et al*. 2007 [9]	Washington State, USA	1999–2003	30 237 (26 270 men, 3967 women), mean age at release: 33.4 years (SD = 9.8) (range = 18–84; with 58% aged 18–34 years).	Time at risk defined as period between release from prison and death, re-incarceration, or end of study. Crude drug-related mortality over a mean follow-up period of 1.9 years was 1.8 per 1000 person-years.
Krinsky *et al*. 2009 [10]	New Mexico State, USA	2001–2003	8380 (men and women—proportions not specified), 10 277 releases: 6600 released once, 1671 released twice, 101 released three times, eight released four times.	Unable to ascertain whether times at risk included re-incarceration or death from other cause, and so periods at liberty may have been slightly overestimated. Estimated person-years from number of releases × assumed days of at-risk period (days halved per deaths), all prior at-risk periods were survived by individuals with multiple releases; no account taken of subsequent incarcerations within 12 weeks after release).

RR: relative risk; SD: standard deviation.

Numbers of drug-related deaths and associated person-years in weeks 1 + 2, 3 + 4 and 5–12 post-release were extracted from eligible studies. When not explicitly available, the author(s) of the study were contacted. Suitable assumptions, stated in footnotes, were made if the data could not meet an aspect of the analysis specification.

### Statistical analyses

We evaluated the RR of drug-related death by calculating the ratio of death rates (deaths relative to person-years) in:

- weeks 1 + 2 versus weeks 3–12, the major comparison in the research literature;
- weeks 1 + 2 versus weeks 5–12, an expansion of the first comparison that enables us to characterize the decay in risk over time; and
- weeks 3 + 4 versus weeks 5–12, to verify and quantify the elevation in risk in weeks 3 + 4 after release.

The associated confidence intervals were calculated as in the Bird & Hutchinson study [3], where the standard error of the logarithm of relative risk was derived as:


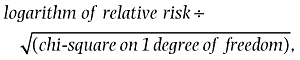


where the χ^2^ test was for homogeneity of risk over time since release.

The heterogeneity between studies was examined using two methods: first, visually by the overlap of the confidence intervals; and then quantitatively by the *I*^2^ statistic [14]. The *I*^2^ statistic measures the impact of heterogeneity in a meta-analysis and is interpreted as the proportion of total variation in the estimates of RR that is due to heterogeneity between studies. The *I*^2^ statistic is preferable to the test for heterogeneity [15,16], which is poorly powered with few studies to support its use [17]. Nevertheless, the *P*-value for this test has been provided here for reference. Based on these assessments, pooled estimates of the RRs were made, where appropriate, using the inverse variance method [18].

We have also analysed the RRs for deaths from all causes within 12 weeks of release. The results for this analysis are not presented in this paper (available from authors).

## RESULTS

Using the above search strategy, we identified 18 potentially relevant studies. Of these, 12 were excluded because:

- studies were of identified drug-users only (Seaman, Brettle & Gore [2]; Shewan *et al*. 2000 [19]; and Christensen *et al*. 2006 [12], who could study only most recent release);
- studies focused upon deaths rather than the follow-up of ex-prisoners after release (Harding-Pink, 1990 [20]; Seymour, Oliver & Black, 2000 [21]; Sattar, 2003 [22]]), with one study [22] having considered mainly deaths of offenders serving in the community;
- two further studies, in addition to Christensen *et al*. [13], referred to ex-prisoners' most recent release (Stewart *et al*. 2004 [23]; Rosen, Schoenbach & Wohl [24]). Fortunately, for one of these studies [23] there was a larger, eligible study at the same facility with an unbiased definition of eligible release (Hobbs *et al*. 2006 [7]); or
- the necessary data were not published, and we were unable to obtain them by contacting the author (Joukamaa, 1998 [25]; Graham, 2003 [11]; Spaulding, Allen & Stone, 2007 [26]). For two of these studies [25,26], further examination revealed that time was measured from the date of prisoners' receptions rather than their date of release (M. Joukamma and A. Spaulding, personal communication, 2008 and 2007, respectively).

**Figure 1:**
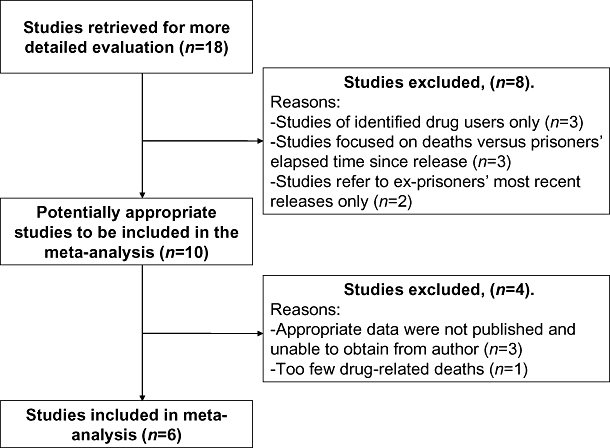
Quality of Reporting of Meta-analyses (QUOROM) diagram of studies included and excluded from meta-analysis

The final exclusion was on grounds of inadequate power: Verger, 2003 [27] reported only one drug-related death in the first 12 weeks after 1305 prisoner releases. Figure 1 presents a QUOROM (Quality of Reporting of Meta-analyses; Moher *et al*. 1999 [28]) diagram of this process.

Six studies were included in the final analysis [3–10] as shown in Table 1.

For the six eligible studies, drug-related causes accounted for 59% of deaths (612 of 1033) within 3 months of release, and 76% within 2 weeks of release (314 of 411) (Krinsky *et al*. [11] identified only ‘deaths in the state that are sudden, unexpected, violent, or untimely, or where a person is found dead with an unknown cause of death’). In total, drug-related deaths versus other deaths were: 314 versus 97 in the first two weeks and 298 versus 327 in the subsequent period.

**Figure 2:**
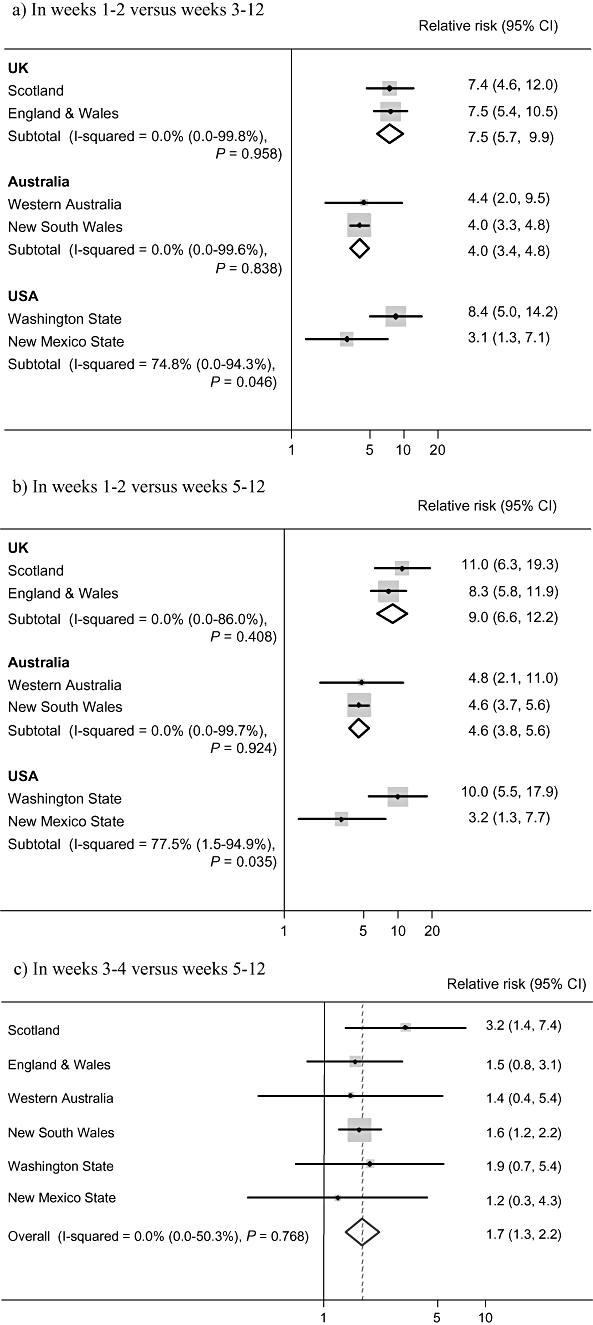
Forest plot of relative risks of drug-related death. (a) In weeks 1–2 versus weeks 3–12. (b) In weeks 1–2 versus weeks 5–12. (c) In weeks 3–4 versus weeks 5–12; CI: confidence interval

Table 2 presents the meta-analyses. For each of the eligible studies, we provide the numbers of drug-related deaths and person-years at risk in weeks 1 + 2, 3 + 4 and 5–12 (13 [4–6]) after release from prison. Alongside, we have the calculated RRs with 95% confidence intervals. RRs for weeks 1 + 2 versus weeks 3–12 were not pooled to give an overall estimate. The non-overlapping confidence intervals and large *I*^2^ statistic indicate that the majority of variation [74%, 95% confidence interval (CI): 40–88%] in the respective RR estimates is explained by heterogeneity between studies. Within the United States, the two studies were also considerably different from each other (*I*^2^ = 75%, 95% CI: −94%). However, pooled RRs, within countries, for the United Kingdom and Australia were appropriate and were estimated. The RRs for weeks 1 + 2 versus weeks 5–12 were pooled similarly. The RRs for weeks 3 + 4, versus weeks 5–12, were sufficiently homogeneous that an overall international estimate could be made. Figure 2 presents the corresponding forest plots.

**Table 2:** Meta-analysis of drug-related deaths in the first 12 (13 weeks for Farrell & Marsden [4,5]) weeks post-release from prison.a

	*Drug-related deaths and person-years (pys) post-release*						*Relative risk (95% CI)*		
	*Weeks 1* + *2*		*Weeks 3* + *4*		*Weeks 5–12*				
*Study*	*Deaths*	*pys*	*Deaths*	*pys*	*Deaths*	*pys*	*Weeks 1* + *2 versus weeks 3–12*	*Weeks 1* + *2 versus weeks 5–12*	*Weeks 3* + *4 versus weeks 5–12*
**United Kingdom**									
Scotland	33b (34)	720b	9b (11)	680b	10b (12)	2 397b	7.4 (4.6–12.0)	11.0 (6.3–19.3)	3.2 (1.4–7.4)
Bird & Hutchinson 2003 [3]	46 per 1000 pys		16 per 1000 pys		5 per 1000 pys				
England and Wales	59	1868	11	1866	32c	8 392c	7.5 (5.4–10.5)	8.3 (5.8–11.9)	1.5 (0.8–3.1)
Farrell & Marsden 2005, 2008 [4–5]	32 per 1000 pys		6 per 1000 pys		4 per 1000 pys				
Interim totals	92	2588	20	2546	42	10 789	**Pooled**: 7.5 (5.7–9.9)	**Pooled**: 9.0 (6.6–12.2)	**Pooled**: 2.0 (1.2–3.5)
	36 per 1000 pys		8 per 1000 pys		4 per 1000 pys				
**Australia**									
Western Australia	10	484	3	477	8	1 842	4.4 (2.0–9.5)	4.8 (2.1–11.0)	1.4 (0.4–5.4)
Hobbs *et al*. 2006 [7]	21 per 1000 pys		6 per 1000 pys		4 per 1000 pys				
New South Wales	177	7275	61	6939	136	25 492	4.0 (3.3–4.8)	4.6 (3.7–5.6)	1.6 (1.2–2.2)
Kariminia *et al*. 2007 [8]	24 per 1000 pys		9 per 1000 pys		5 per 1000 pys				
Interim totals	187	7759	64	7416	144	27 334	**Pooled**: 4.0 (3.4–4.8)	**Pooled**: 4.6 (3.8–5.6)	**Pooled**: 1.6 (1.2–2.2)
	24 per 1000 pys		9 per 1000 pys		5 per 1000 pys				
**United States**									
Washington State	27	1466	5	1426	10	5 409	8.4 (5.0–14.2)	10.0 (5.5–17.9)	1.9 (0.7–5.4)
Binswanger *et al*. 2007 [9]	18 per 1000 pys		4 per 1000 pys		2 per 1000 pys				
New Mexico State	8	394	3	394	10	1 573	3.1 (1.3–7.1)	3.2 (1.3–7.7)	1.2 (0.3–4.3)
Krinsky *et al*. 2009 [10]	20 per 1000 pys		8 per 1000 pys		6 per 1000 pys				
Heterogeneity measure: *I*^2^ statistic (95% CI) Cochran's χ^2^ test *P*-value							74% (40–88%) *P* = 0.002	74% (39–89%) *P* = 0.002	0% (0–50%) *P* = 0.768

a Both sexes combined for all except Scotland (males only).

b Using authors' method A: censors at earlier of first re-incarceration or death.

c Next 9 weeks (versus next 8 weeks). CI: confidence interval.

The risk of drug-related death was at least threefold during weeks 1 + 2 compared with weeks 3–12. The pooled RR for UK studies was the highest: 7.5 (95% CI: 5.7–9.9), and the lowest was for the Australian studies: 4.0 (95% CI: 3.4–4.8). For the two US studies, the RR of the Washington State study coincided with the UK studies (8.4, 95% CI: 5.0–14.2), while the RR of the New Mexico study was markedly different, but numerically similar to the Australian studies (3.1, 95% CI: 1.3–7.1).

The RRs for weeks 1 + 2 versus weeks 5–12 were ordered similarly, although slightly higher: 9.0 (95% CI: 6.6–12.2) pooled for the United Kingdom and 4.6 (95% CI: 3.8–5.6) pooled for Australia. The RRs for weeks 3 + 4 are more similar and so could be pooled to give an overall, international estimate: 1.7 (1.3–2.2).

While the rates of drug-related death during weeks 1 + 2 varied considerably between eligible studies (18–46 per 1000 person-years), the rates during weeks 5–12 were broadly similar: approximately four per 1000 person-years, albeit slightly lower for Washington State.

## DISCUSSION

### Key findings

This meta-analysis demonstrated an internationally high, three- to eightfold increased risk of drug-related death in the first 2 weeks after release from prison compared with the subsequent 10 weeks. There was heterogeneity in fatal overdose risk across the three continents represented, with one US and the two Australian studies showing a much lower overdose risk in the first 2 weeks post-release than the other US and two UK studies. The risk of drug-related death is also shown to remain elevated beyond the first 2 weeks post-release into the second 2 weeks (relative to weeks 5–12). This reinforces earlier observations [3][4–6] that, while the risk decreases by weeks 3 + 4, it remains importantly raised.

### Interpretation

The increased risk of drug-related death may be explained by a decrease in tolerance to drugs as a result of being in prison where drug use is less frequent and the drugs may be of lower purity [1]; and there could be a tendency for ‘celebration’ on release. Across the six studies the RRs for weeks 1 + 2 are elevated to different extents, which is perhaps unsurprising given the possible variations in prisons' drug policies and in the nature of illicit drug use more broadly. We highlight here potential explanations for the differently elevated RRs in the first 2 weeks.

The purity of heroin (as well as methamphetamines, cocaine and crack cocaine) could vary according to manufacturing and availability; and associations between heroin purity and the occurrence of drug-related deaths have been found [29,30]. The prevalence of injecting versus non-injecting routes of opioid administration may vary regionally, and injection poses the greater overdose risk [31]. There may also be regional variations in the patterns of co-use of alcohol, benzodiazepines and other depressants of the central nervous system. In combination with heroin, such depressants potentially present an increased overdose risk [21,30,34]. Upon release from prison, regional variations in the above drug use behaviours or cultures could contribute to differential effects on the immediate risk of drug-related death, before tolerance is restored.

In addition, drug treatment programmes may differ in availability, both inside and outside prisons; in methadone maintenance dose (>50 mg); and the protection they afford against injection-related risk behaviours and premature mortality [32,33].

Studies also varied by the age, sex and length of incarceration of their respective ex-prisoners. The Scottish study focused upon 15–35-year-old males who had been incarcerated for at least 14 days, to target the younger, short sentence profile of drug-users [3]. In Binswanger *et al*. [10], two-fifths of released prisoners were outside this age range; females were included (3967 of 30 237); and releases were from ‘prisons’ which, by definition in the United States, are for individuals sentenced for more than 1 year (plus those re-incarcerated for violating parole). To try to identify aspects in correctional policies or prisoners' demography that determine their drug-related death risk soon after release, more studies of the type included in this meta-analysis from other correctional systems would be needed.

We note that the large, ineligible, last-release historical study in North Carolina, 1980–2005 [24] yielded a low relative risk comparing weeks 1 + 2 with weeks 3–12, 3.1 (95% CI: 1.7–5.5). This was in agreement with the New Mexico study [10], 3.1 (95% CI: 1.3–7.1), but considerably lower than the study in Washington State [9], 8.4 (95% CI: 5.0–14.2). Given that Rosen, Schoenbach & Wohl [24] introduced upward bias by studying ex-prisoners' most recent incarceration, one might have expected higher absolute risks to be reported.

The Australian studies [7,8] exhibited the lowest RR for weeks 1 + 2. One explanatory factor could be the earlier establishment of methadone maintenance in NSW prisons (readily available for prisoners from the late 1980s [35] versus from only 2003 in Scotland [36]). This was tested in an exploratory re-analysis of the NSW data for consecutive 5-year periods. Unexpectedly, the RR for weeks 1 + 2 actually increased from 2.7 (95% CI: 1.7–4.3) in 1988–92 to 5.1 (95% CI: 3.8–6.9) in 1998–2002. Of course, temporal analyses are liable to confounding. Australia's average heroin purity roughly trebled during 1996–2000 [37], which may have caused an increase in overdose fatalities [29]. Moreover, Australia's heroin drought at the end of 2000 [38] is well known for having complicated the interpretation of its drug-related mortality trends.

Another possible explanation for the observed variation in risks could be studies' different definitions of drug-related death (see Appendix S1). For example, suicides were excluded from the definition of drug-related death adopted by Bird & Hutchinson [3], but included by Farrell & Marsden [4–6] and Kariminia *et al*. [9] (and by Rosen, Schoenbach & Wohl [24]). The impact of these differing definitions was explored by re-analysing the NSW data according to the definition used by Bird & Hutchinson [3]. This yielded virtually unaltered RRs (data available from authors), suggesting that different definitions did not account for the heterogeneity. Nevertheless, studies' different definitions remain an acknowledged limitation of our meta-analysis.

Inclusion of the study by Verger *et al*. [27] would have caused analytical problems because they did not observe any drug-related deaths in weeks 1 + 2 and only one in weeks 3–12 after release. Under the null hypothesis of homogeneity of risk over the 12-week period, the expected numbers of such deaths in weeks 1 + 2 would be so small that the large sample approximation, upon which the χ^2^ distribution is based, breaks down. In our view, including such studies does not make statistical sense and we suggest that meta-analyses more generally may benefit from the exclusion of such poorly powered studies from the outset.

This meta-analysis includes only six studies, but each is moderately powerful and they conform to robust eligibility criteria. As forewarned by Seaman *et al*. [2], our 12-week follow-up period avoids major confounding by periodic changes in ex-prisoners' drug use. The findings do not rely on either ex-prisoners' willingness to self-identify as drug-using or the diagnosis of drug dependence. Analyses are unbiased and estimates have been pooled only where appropriate. The meta-analysis was restricted to studies with associated publications in the English language, so it is possible that studies in other languages were missed. However, as the search retrieved studies from Finland, Denmark and France we hope that studies omitted by restricting to English-language studies are few, if any. Further studies from resource-poor countries and other continents would, however, be highly instructive.

Future studies of drug-related death soon after release from prison should be well designed, along the lines of the eligibility criteria for this meta-analysis. For sufficient statistical power, we suggest that studies should be of at least 10 000 index releases (10 000 unique ex-prisoners), with at least eight drug-related deaths in the first 2 weeks. Based on the calculations of Bird & Hutchinson [3], studies of this size would have at least 50% statistical power to detect a RR of 4 for drug-related death during weeks 1 + 2 versus weeks 3–12 after release from prison.

Remedial action is required by both prisons and communities to address this observed elevation in risk. Prisoners should be alerted to the high risk of overdose death soon after release, advised not to be alone if they use drugs and to be wary of mixing heroin with other drugs, including alcohol [34]. Transitional care programmes, which provide pre- and post-release treatment and support, are promising interventions but, as yet, the research has not been conclusive. Novel programmes include prison-based education about naloxone (heroin antidote) and its prescription to prisoners with a history of heroin injection [3].

## CONCLUSION

In conclusion, further research is needed urgently on mortality after release from prison, as well as interventions to reduce the risk of drug-related death during the transition from prison to the community. At present, the regional and cultural variations in drug use limit what can be learnt from the strategies and experiences of other countries around the world. With suitable data from additional studies, we may be able to identify key characteristics of regions, prisons or prisoners that explain the variation in the RR of drug-related death soon after release. Nevertheless, the elevation in risk clearly exists, and findings from pharmaceutical experimental studies of remedial interventions are likely to be transferable between countries.

## Supporting information

**Appendix S1.** Definition of drug-related death compared across studies of prisoners-on-release.
