# Supplementary material for: Meta-analysis of drug-related deaths soon after release from prison
Source: Addiction. 2010 Sep;105(9):1545–54. doi: 10.1111/j.1360-0443.2010.02990.x (PMC2955973; doi:10.1111/j.1360-0443.2010.02990.x)
Supplement: Supplementary file 1 [file add0105-1545-SD1.doc]

**APPENDIX S1**

Definition of Drug-related Death Compared Across Studies of Prisoners-on-release.

| **ICD-10 code** | **ICD-9 code** | **Description** | **Farrell**  **& Marsden, 2008** | **Bird &**  **Hutchinson, 1998** | **Hobbs *et al.* and Kariminia *et al.* 2006 & 2007** | **Binswanger**  ***et al.* 2007** | **Krinsky *et al.[[1]](#footnote-2)* 2009** |
| --- | --- | --- | --- | --- | --- | --- | --- |
| F11-F19  excl. F17 | 292 | Drug-induced mental disorders | X |  |  |  |  |
|  | 304 excl 304.6 | Drug dependence | X | X | X |  |  |
|  | 304.6 | Drug dependence (solvent) | X |  | X |  |  |
| Above + F55 | 305.2– 305.9 | Non-dependent abuse of drugs | X |  | X |  |  |
| X40-X44 | E850- E858 | Accidental poisoning by drugs, medicaments & biological substances | X | X | X | X | X |
| X45-X49 | E860- E869 | Accidental poisoning by other solid & liquid substances, gases & vapours |  |  |  | X | X |
| X60-X64 | E950.0-E950.5 | Intentional self-poisoning by drugs, medicaments & biological substances | X |  | X |  |  |
| X85 | E962.0 | Assault by poisoning – drugs and medicinal substances |  |  | X |  |  |
| Y10-Y14 | E980.0-E980.5 | Poisoning by drugs, medicaments & biological substances, undetermined intent | X | X | X |  |  |
| Y15-Y19  excl. Y17 | E980.6-E980.9 | Poisoning by other solid & liquid substances, gases & vapours, undetermined intent |  | X |  |  |  |

1. Inferred from published article: “deaths caused by drug overdose and classified as accidental in manner”. Unable to obtain a more precise definition from the authors. [↑](#footnote-ref-2)
